# Rural-urban disparities in basic sanitation access among households: a multivariable decomposition analysis of Ethiopian demographic and health survey 2019

**DOI:** 10.3389/fpubh.2024.1420077

**Published:** 2024-11-01

**Authors:** Awoke Keleb, Chala Daba, Abel Endawkie, Lakew Asmare, Fekade Demeke Bayou, Eyob Tilahun Abeje, Aznamariam Ayres, Anissa Mohammed, Natnael Kebede, Kaleab Mesfin Abera, Asnakew Molla Mekonen, Endalkachew Mesfin Gebeyehu, Shimels Derso Kebede, Ermias Bekele Enyew, Mastewal Arefaynie, Abiyu Abadi Tareke, Yawkal Tsega

**Affiliations:** ^1^Department of Environmental Health, College of Medicine and Health Sciences, Wollo University, Dessie, Ethiopia; ^2^Department of Epidemiology and Biostatistics, School of Public Health, College of Medicine and Health Sciences, Wollo University, Dessie, Ethiopia; ^3^Department of Health Promotion, School of Public Health, College of Medicine and Health Sciences, Wollo University, Dessie, Ethiopia; ^4^Department of Health System and Management, School of Public Health, College of Medicine and Health Sciences, Wollo University, Dessie, Ethiopia; ^5^Department of Health Informatics, School of Public Health, College of Medicine and Health Sciences, Wollo University, Dessie, Ethiopia; ^6^Department of Reproductive and Family Health, School of Public Health, College of Medicine and Health Sciences, Wollo University, Dessie, Ethiopia; ^7^AMREF Health in Africa, COVID-19 Vaccine/EPI Technical Assistant at West Gondar Zonal Health Department, Gondar, Ethiopia

**Keywords:** basic sanitation service, decomposition, EDHS, Ethiopia, improved, rural–urban

## Abstract

**Introduction:**

Disparities in access to basic sanitation services between rural and urban households pose significant challenges to public health and human development. Understanding the determinants contributing to this gap is vital for advancing the Sustainable Development Goals (SDGs) and improving environmental and public health through evidence-based interventions.

**Objective:**

This study aims to analyze and understand the disparities in access to basic sanitation services between rural and urban households in Ethiopia.

**Methods:**

This study analyzed a sample of 8,663 weighted households, collected using stratified sampling techniques, utilizing the data from the 2019 Ethiopian Demographic and Health Survey (EDHS). The primary outcome was basic sanitation access, defined as access to flush or pour-flush systems, septic tanks, pit latrines, or composting toilets. A multivariable decomposition analysis was conducted to identify factors contributing to rural–urban disparities. Statistically significant variables were determined at a *p*-value of <0.05 with a 95% confidence interval.

**Results:**

The weighted proportion of basic sanitation access in Ethiopia was 13.78% (95% CI, 12.67–14.96), with significant disparities between rural (6.02%) and urban (27.15%) residents. Endowment factors accounted for 78.9% of this disparity, while behavioral coefficients contributed 22.1%. If the characteristics of respondents in rural and urban households had been similar, significant factors that would have narrowed the gap included the age of the household head (15–35 years), the absence of under-five children, smaller family size, and the attainment of secondary education, reducing the gap by 1.83, 2.07, 5.08, and 3.25%, respectively. Conversely, illiteracy and primary education levels widened the gap between rural and urban access to basic sanitation services by 16.85 and 0.23%, respectively. Additional factors exacerbating the rural–urban disparity included poverty (which widened the gap by 58.71%), residence in pastoralist regions (which widened the gap by 10.10%) or agrarian regions (which widened the gap by 7.03%), and access to water sources located more than 30 min away (which widened the gap by 7.91%).

**Conclusion:**

Significant disparities in access to basic sanitation services exist between rural and urban households in Ethiopia. Key factors contributing to these disparities include the age of the household head, education level, family size, region of residence, and water source proximity. Addressing these factors is essential for improving sanitation access and achieving the Sustainable Development Goals (SDGs).

## Introduction

Access to basic sanitation services remains a critical global concern, as it directly impacts public health outcomes, environmental sustainability, and human dignity (1). Target 6.2 of the Sustainable Development Goals (SDGs) specifically calls for universal access to adequate and equitable sanitation (2). A key indicator for this target is the proportion of the population with access to basic sanitation services, with a focus on equity and quality, including disparities based on geography, socioeconomic status, gender, and other demographic factors (1, 3, 4).

According to the Joint Monitoring Report 2021, approximately 71% of countries have achieved universal access to basic sanitation services (3, 4). However, despite significant progress in recent years, access to basic sanitation remains a critical challenge globally, especially in low- and middle-income countries, where significant disparities persist between urban and rural areas (4, 5).

Approximately 3.6 billion individuals lack access to safely managed sanitation services, including 1.9 billion with only basic services, 580 million with limited services, 616 million relying on unimproved facilities, and 494 million practicing open defecation. Two-thirds of those without access to even basic sanitation services reside in rural areas. Nearly half of these individuals live in sub-Saharan Africa, with 92% of the population practicing open defecation and residing in rural areas (3).

In low-income countries, 62% of urban residents live in poor sanitation conditions (6, 7). In sub-Saharan Africa, only 4% of the rural population and 23% of the urban population have access to basic sanitation services (3). According to the 2019 Ethiopian Demographic and Health Survey, the overall proportion of households with access to basic sanitation services varies significantly, ranging from 6% in Somali to 49% in Addis Ababa. Additionally, 20% of households in Ethiopia use improved toilet facilities, with 42% in urban areas and just 10% in rural areas. Over half (56%) of rural households use unimproved toilet facilities, and more than one in four households (27%) in Ethiopia has no toilet facility at all, with the disparity being 35% in rural areas and 10% in urban areas (8).

While numerous studies in Ethiopia have investigated the factors associated with access to improved sanitation facilities, a systematic review and meta-analysis (6) was conducted on the spatial distribution of household access to improved sanitation facilities and its associated factors in Ethiopia. However, none of them have specifically addressed the endowment factors contributing to disparities in access to basic sanitation services and covariates between rural and urban areas.

As a result, there is a lack of scientific data regarding the percentage contribution of influencing factors that account for the differences in household access to basic sanitation services between rural and urban areas in Ethiopia. These data are crucial for developing evidence-based interventions and strategies. Therefore, a decomposition analysis is required to identify the specific coefficients and percentage contribution through which these factors influence rural–urban disparities (9, 10). This analysis includes investigating the interplay between wealth status, educational status, gender inequality, region of residence, proximity to water sources, and access to basic sanitation services in both rural and urban settings.

Therefore, the primary objective of this study is to analyze and understand the urban–rural disparities in access to basic sanitation services using the data from the 2019 Ethiopian Mini-Demographic and Health Survey. The findings of this study are significant, as they provide valuable insights for informing targeted interventions and designing evidence-based strategies to enhance public and environmental health outcomes. Additionally, these findings can guide policy decisions to promote healthier communities, achieve the SDGs, and reduce inequalities in accessing basic sanitation services.

## Methods

### Study design, setting, and period

We used data from the 2019 Ethiopian Mini-Demographic and Health Survey (EMDHS), which is available at the https://www.dhsprogram.com/data/dataset_admin/login_main.cfm website. From 21 March 2019 to 28 June 2019, a community-based cross-sectional study was conducted in rural and urban areas of Ethiopia as part of the second round of the EMDHS. The first round of the survey was conducted in 2014.

Ethiopia, located in the Horn of Africa, lies between latitudes 3° and 15° N and longitude 48° E. It covers a vast area of 1,100,000 square km and is divided into 11 ethnically and politically autonomous regional states along with two administrative cities. Over time, Ethiopia has experienced significant population growth, escalating from 53.5 million during the 1994 census to a staggering 114,968,588 in 2020, with a fertility rate of 4.3.

### Population and eligibility criteria

The source population for this study included all households in Ethiopia, while the study population comprised households from selected enumeration areas.

### Sample size and sampling techniques

A sample size of 8,663 weighted households was used for this study. Sampling weights were applied to ensure representativeness and to address non-proportional allocation across regions, rural–urban differences, and potential response rate variations. A total of 21 sampling strata were established for the study. Initial sampling units were enumeration areas (EAs), with 25 EAs selected from 8 regions using an equal allocation method to ensure survey precision consistency (11).

Larger regions such as Amhara, Oromia, and the Southern Nations, Nationalities, and Peoples’ Region (SNNPR) each had 35 EAs. In total, 305 EAs (212 rural and 93 urban EAs) were selected with probabilities proportional to EA size, based on the 2019 Ethiopian Population and Housing Census (EPHC) frame (12, 13).

Sampling occurred in two stages: first, stratified samples of census EAs in rural and urban zones were selected, followed by systematic probability sampling of households within these EAs (11). Household heads were interviewed using an individual questionnaire.

### Data collection tools and variables

The 2021 WHO and UNICEF indicators for assessing the sanitation service ladder were used to measure access to basic sanitation services (3). A household is considered to have access to basic sanitation if it has an unshared flush or pour-flush toilet connected to a piped sewer system, a septic tank, or pit latrines or if it uses a ventilated improved pit latrine, composting toilet, or pit latrine with a slab and does not know where they disposed their excreta. Households were classified as having unimproved sanitation services if they had any of the following toilet types: flush toilets that discharge elsewhere, pit latrines without slabs, open pits, buckets, hanging toilets, or practiced open defecation (including no facility, bush, or field). These conditions were categorized as poor sanitation service access (3, 14).

Access to basic sanitation services, categorized as urban and rural, was the main predictor variable in this study. Explanatory factors included the age of the household head, educational status of the household head, sex of the household head, number of family members, presence of under-five children in the household, proximity to water sources, and media exposure. The data were collected through face-to-face interviews with the household heads.

### Data management and analysis

The data were cleaned, labeled, processed, and analyzed using STATA v17.0. Weighted frequencies and percentages were computed to address the non-response rate and design effect of DHS data. Descriptive statistics, including frequencies with percentages, means, and standard deviations, were reported, and the results are shown in tables and graphs.

Coefficients with 95% confidence interval and a *p*-value of 0.05 were utilized to establish statistical significance. A multivariable Oaxaca decomposition analysis was conducted to analyze rural–urban disparities in access to basic sanitation services among households in Ethiopia. This method dissects the output of regression models, such as means or proportions, into components that are attributable to compositional differences between groups.

The rural–urban disparities in access to basic sanitation services were decomposed into the endowment effect (contribution of respondents’ characteristics and their environment), the coefficient effect (the response to behavior), and the interaction of the two. The difference in access to basic sanitation services was ascribed to a gap in endowments (E), coefficients (C), or the interaction of endowments and coefficients. The Oaxaca decomposition uses the high group (urban household) as the reference group, with weighting contrasts in attributes by the coefficients of urban children and contrasts in coefficients by the covariates of rural children (15, 16).

If Yi is the outcome variable and an X is an independent variable with two groups, rural and urban, then access to basic sanitation service for the rural and urban households is represented as *ε*.


$${Y_{i}}^{\mathrm{rural}}=\beta^{\mathrm{rural}}X_{i}+\varepsilon^{\mathrm{rural}}\text{.}$$

$${Y_{i}}^{\mathrm{urban}}=\beta^{\mathrm{urban}}X_{i}+\varepsilon^{\mathrm{urban}}\text{.}$$

Thus, the rural–urban disparities in the mean access to basic sanitation service (Y^rural^ – Y^urban^) is given as follows:


$$\begin{array}{l} Y^{\mathrm{rural}}–Y^{\mathrm{urban}}=\left(X^{\mathrm{rural}}–X^{\mathrm{urban}}\right)\;\beta \mathrm{urban}+\left(\beta^{\mathrm{rural}}–\beta^{\mathrm{urban}}\right)\;\\ X^{\mathrm{rural}}+\left(X^{\mathrm{urban}}-X^{\mathrm{rural}}\right)\left(\beta^{\mathrm{urban}}–\beta^{\mathrm{rural}}\right)\text{.} \end{array}$$

$$Y^{\mathrm{rural}}–Y^{\mathrm{urban}}=\Delta X\beta^{\mathrm{rural}}+\Delta \beta X^{\mathrm{urban}}+\Delta X\Delta \beta \text{.}$$

=E+C+CE,

where ΔX represents the mean difference in explanatory variables (X^rural^ – X^urban^).

## Results

### Sociodemographic characteristics

As outlined in the EDHS 2019 mini-report, there are significant socioeconomic and demographic disparities between rural and urban households. This study included a total of 8,663 weighted participants. The mean age of the household heads was 43.05 years (SD = 16.49). Among them, 2,343 (38.93%) rural and 1,252 (47.33%) urban household heads were in the 15–35 year age group. In rural areas, a significant proportion of household heads were over 50 years of age, accounting for 1,770 (73.35%) households, while in urban areas, the proportion was lower at 643 (26.65%).

Moreover, educational attainment varied significantly, with 3,443 (83.41%) rural household heads lacking formal education, compared to only 685 (16.59%) in urban areas. Male household heads were more prevalent in rural settings, representing 4,606 (73.22%) of the households, compared to 1,685 (26.78%) in urban areas. Rural households were generally larger, with 2,413 (79.82%) having more than five members, whereas urban households had a lower proportion at 610 (20.18%). Additionally, the presence of under-five children was higher in rural areas (3,261, 75.43%) compared to urban areas (1,062, 24.57%). Regional differences also emerged, with a higher concentration of agrarian communities in rural areas (3,210, 85.46%) compared to pastoralist communities (2,254, 82.02%).

Furthermore, wealth distribution highlights a stark contrast between rural and urban areas, with rural regions characterized by a majority of poor households (3320) (94.91%), while urban areas have a higher proportion of rich households (2,413) (62.19%). Access to basic amenities, such as water sources, is also significantly less convenient in rural areas, where 1,749 households (83.09%) take more than 30 min to access water, compared to just 356 households (16.91%) in urban settings.

Similarly, 1,455 rural (61.47%) households lack media exposure, compared to 912 households (38.53%) in urban areas (Table 1). These findings highlight the significant socioeconomic and demographic disparities between rural and urban areas, highlighting the need for targeted interventions to address these disparities and promote equitable development.

**Table 1:** Socio-economic and demographic characteristics, other explanatory variables of household participants based on place of residence using EDHS 2019 mini report.

Variables	Category	Rural	Urban
Age of the of household head	15–35	2,343 (65.17%)	1,252(34.83%)
	36–50	1905 (71.75%)	750 (28.25%)
	>50	1770 (73.35%)	643 (26.65%)
Educational status of household head	Unable to read and write	3,443 (83.41%)	685 (16.59%)
	Primary education	1909 (70.31%)	806 (29.69%)
	Secondary education	398 (41.33%)	565 (58.67%)
	College and above	268 (31.27%)	589 (68.73%)
Sex of household head	Male	4,606 (73.22%)	1,685(26.78%)
	Female	1,412 (59.53%)	960 (40.47%)
Family size	≤5 members	3,605(63.92%)	2035 (36.08)
	>5 members	2,413(79.82%)	610 (20.18%)
Presence of under 5 children	No	2,757 (63.53%)	1,583(36.47%)
	Yes	3,261 (75.43%)	1,062(24.57%)
Region	Pastoralist	2,254 (82.02%)	494(17.98%)
	Agrarian	3,210 (85.46%)	546(14.54%)
	Urban	554 (25.66%)	1,605(74.34%)
Wealth index	Poor	3,320 (94.91%)	178 (6.7%)
	Middle	1,231 (95.80%)	54 (4.20%)
	Rich	1,467 (37.81%)	2,413(62.19%)
Proximity to water source	Takes >30 min	1749 (83.09%)	356 (16.91%)
	Takes ≤30 min	4,269 (65.10%)	898 (34.90%)
Access to media exposure	No	1,455 (61.47%)	912 (38.53%)
	Yes	4,563 (72.47%)	1733(27.53%)

### Proportion of access to basic, limited, improved, and open defecation

Rural areas exhibited lower access to basic sanitation services than urban regions, indicating that 372 (6.18%) rural households and 730 (27.60%) urban households had access to basic sanitation services. Similarly, fewer households, with 232 (3.86%) in rural areas and 1,044 (39.47%) in urban households, had limited sanitation services.

The largest disparity was observed in unimproved sanitation services, with 2,806 (46.63%) rural households and 636 (24.05%) urban households lacking sanitation services.

**Figure 1:**
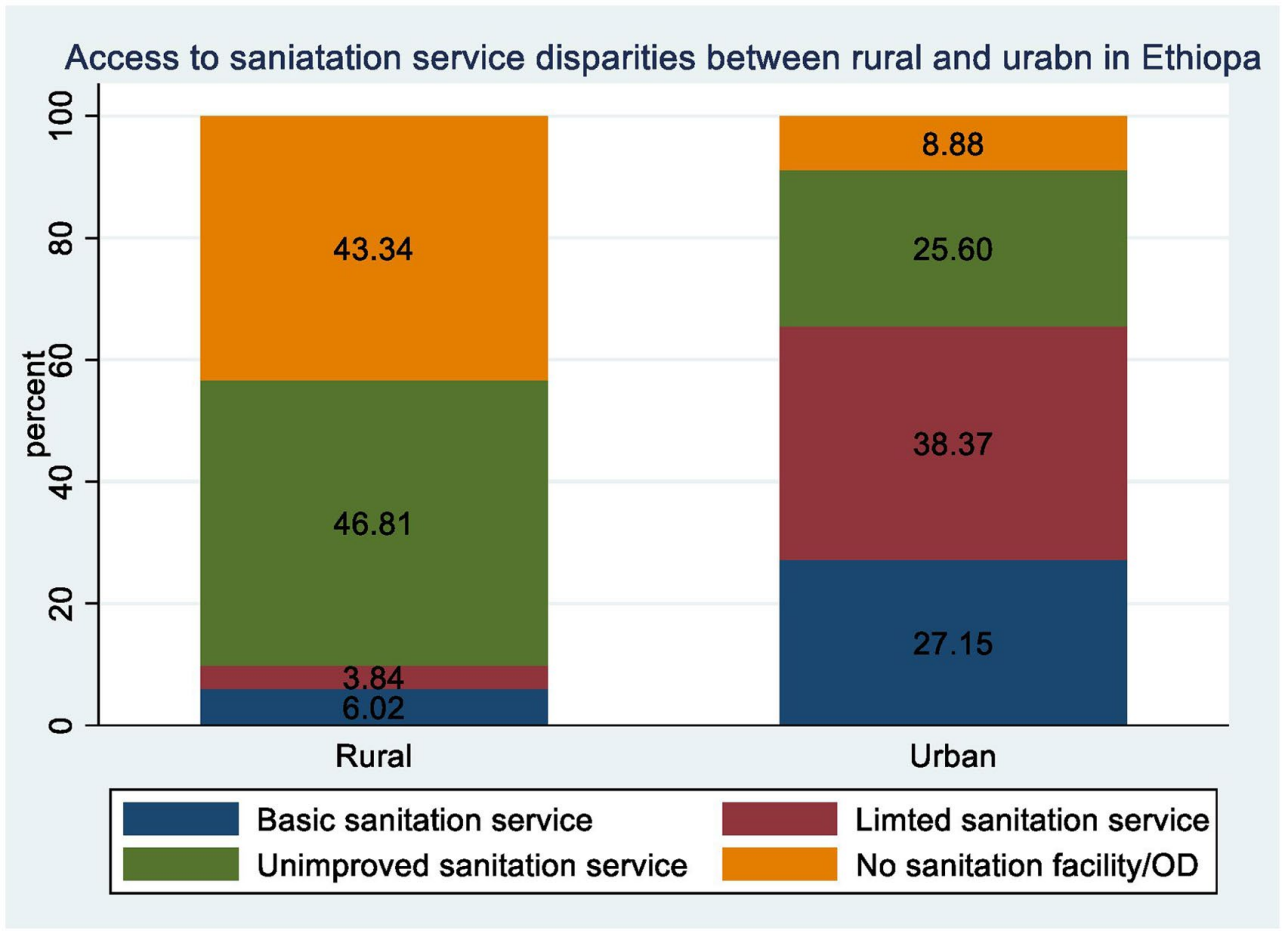
Rural–urban disparity of access to basic, limited, improved and absence of sanitation services among household in Ethiopia from EDHS 2019.

**Figure 2:**
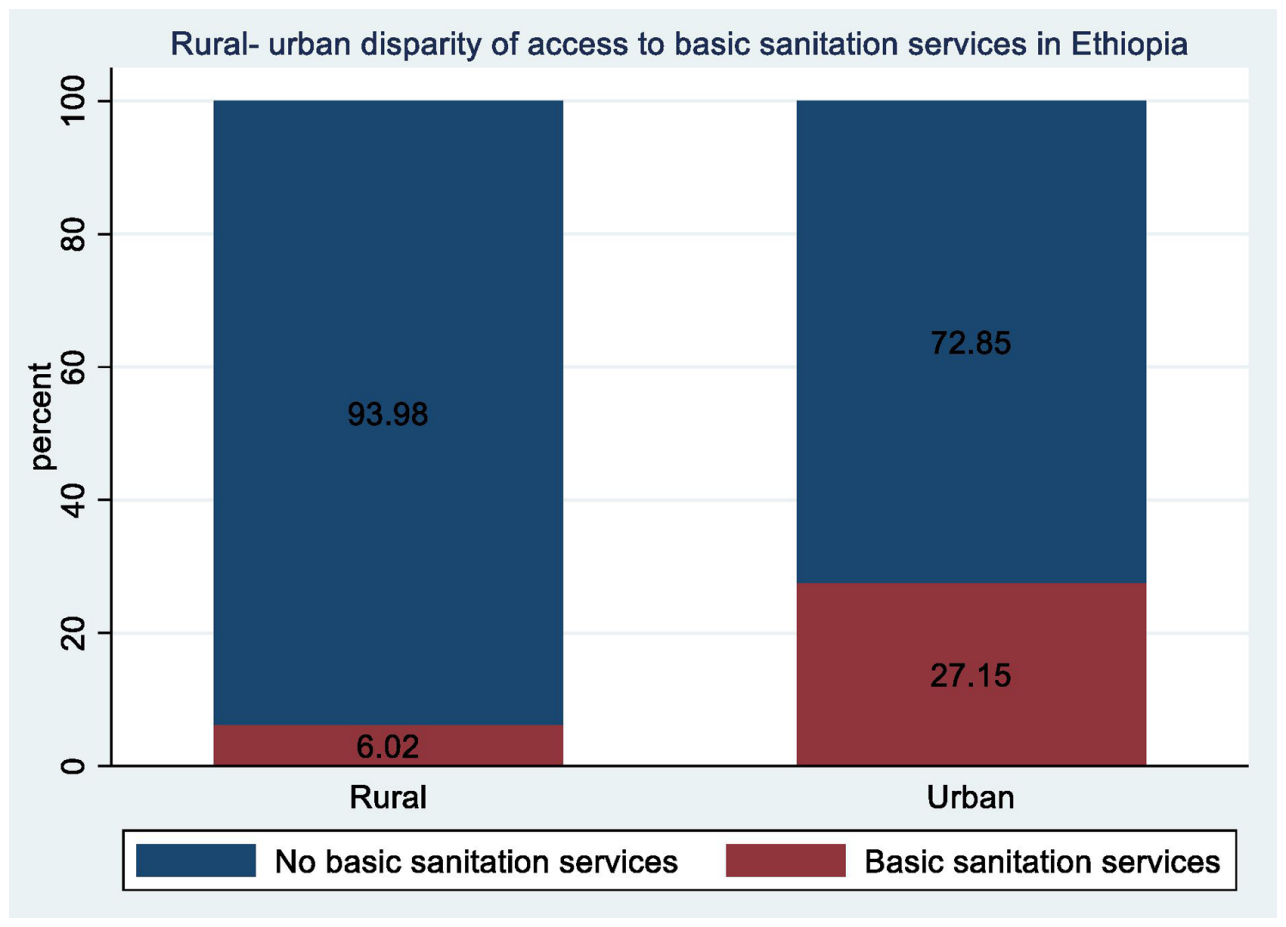
Rural-urban disparity of access to basic sanitation services among household in Ethiopia from EDHS 2019.

Finally, the absence of sanitation facilities was notably higher in rural areas, with 2,608 (43.34%) rural households lacking sanitation facilities or practicing open defecation, compared to only 235 (8.88%) urban households (Figure 1). These disparities underscore the urgent need for targeted interventions to improve sanitation infrastructure, particularly in rural areas, to ensure equitable access to essential services for all communities (Figure 2).

Access to basic sanitation services was disaggregated by region, indicating that 2,748 households in the pastoralist region, of which 2,608 (94.91%) residents have no basic sanitation service, and 140 (5.09%) have basic sanitation service. From a total of 3,756 households in the agrarian region, 3,445 (91.72%) residents have no basic sanitation service, whereas 311 (8.28%) have basic sanitation service. Out of 2,159 households in the urban region, including Addis Ababa, 295 (42.02%), only 651 (30.15%) have access to basic sanitation services.

Overall, the weighted proportion of households with access to basic sanitation services in Ethiopia was 13.78% (95% CI, 12.67–14.96), while 86.22% (95% CI, 85.03–87.32) did not have access to such services (limited, unimproved, or no sanitation services). The proportion of access to basic sanitation services among rural residents was much lower than that among urban residents, with 6.02 and 27.15%, respectively (Figure 2).

### Decomposition analysis

Tables 2, 3 show the decomposition analysis. There was a significant difference or disparity in access to basic sanitation service between rural and urban households, in which rural residents had 0.22 times lower access to basic sanitation service than urban residents. This disparity was explained mostly due to difference in endowment characteristics with 78.9% (*p* value <0.001). This indicates, if the respondents’ characteristics in rural and urban households had been similar, the gap related to access to basic sanitation services would have decreased by 78.9%.

**Table 2:** Endowment factors of decomposition analysis of rural urban disparity of access to basic sanitation service among household in Ethiopia from EDHS 2019.

	Coefficient with 95%CI	Percent	*p*-value
Raw difference	0.28 (0.251–0.302)	100	<0.001
Explained	0.22 (0.184–0.253)	78.9	<0.001
Unexplained	0.06 (0.025–0.091)	22.1	0.001

	Endowment (explained characteristics)		
	Coefficient (95% CI)	Percent	*P* value
Age of household head			
15–35	−0.00505 (−0.00680- −0.00329)	−1.83	**<0.001**
36–50	0.00047 (−0.00025–0.00120)	0.17	0.203
>50	1		
Sex of household head			
Male	−0.00070 (−0.00304–0.00164)	−0.25	0.560
Female	1		
Family size			
≤5 members	−0.01405 (−0.02107–0.00704)	−5.08	**<0.001**
>5 members	1		
Educational status of household head			
Unable to read and write	0.04657 (0.02953–0.06360)	16.85	**<0.001**
Primary education	0.00064 (0.00041–0.00088)	0.23	**<0.001**
Secondary education	−0.00899 (−0.01461- −0.00337)	−3.25	**0.002**
College and above	1		
Presence of under five children			
No	−0.00574 (−0.01052- −0.00095)	−2.07	**0.019**
Yes	1		
Region			
Pastoralist	0.02793 (0.00700–0.04886)	10.10	**<0.001**
Agrarian	0.01942 (0.01171–0.02714)	7.03	**<0.001**
Urban	1		
Wealth status			
Poor	0.16227 (0.12056–0.19294)	58.71	**<0.001**
Medium	0.01759 (−0.00761–0.04475)	6.36	0.163
Rich	1		
Proximity to water source			
Takes >30 min	−0.02185 (−0.03381- −0.00989)	−7.91	**<0.001**
Takes ≤30 min	1		
Media exposure			
No	−0.00037 (−0.00327–0.00252)	−0.15	0.801
Yes	1		

The bold indicates statistically significant variables (*p* < 0.05) for disparities in basic sanitation access between rural and urban household.

**Table 3:** Coefficient (behavioral) factors of the decomposition Analysis of rural urban disparity of access to basic sanitation service among household in Ethiopia from EDHS 2019.

	Due to difference in Coefficient (Unexplained)		
	Coefficient (95% CI)	Percent	*P* value
Age of household head			
15–35	−0.03554 (−0.11689–0.04580)	−12.86	0.392
36–50	0.01550 (−0.02660–0.05761)	5.61	0.471
>50	1		
Sex of household head			
Male	−0.00647(−0.08378–0.07084)	−2.34	0.870
Female	1		
Family size			
≤5 members	−0.08874 (−0.25575–0.07826)	−32.11	0.298
>5 members	1		
Educational status of household head			
Unable to read and write	−0.15731 (−0.45010–0.13549)	−56.91	0.292
Primary education	−0.06466 (−0.19036–0.06105)	−23.39	0.313
Secondary education	−0.00802 (−0.02798–0.01194)	−2.90	0.431
College and above	1		
Presence of under five children			
No	0.01420(−0.03270–0.06110)	5.14	0.553
Yes	1		
Region			
Pastoralist	0.07615 (−0.06616–0.21847)	27.55	0.294
Agrarian	−0.02430 (−0.09399–0.04539)	−8.79	0.494
Urban	1		
Wealth status			
Poor	−0.21217 (−0.67590–0.25156)	−76.76	0.370
Medium	−0.01359 (−0.07485–0.04768)	−4.92	0.664
Rich	1		
Proximity to water source			
Takes >30 min	0.04298 (−0.04272–0.12868)	15.55	0.326
Takes ≤30 min	1		
Media exposure			
No	−0.00440 (−0.02648–0.01768)	−1.59	0.696
Yes	1		

The change in the effect of endowment characteristics among households contributing to the rural–urban disparities in access to basic sanitation services includes factors such as the age of the household head (15–35 years), households with five or fewer members, inability to read and write, attainment of primary and secondary education, the presence of under-five children, residence in pastoralist and agrarian regions, poverty, and taking more than 30 min to access water sources. These factors significantly contributed to the observed disparities in access to basic sanitation services.

Difference in wealth status (being poor) between rural and urban households was the primary factor, accounting for 58.71% (*β* = 0.16227, 95%CI: 0.12858–0.19596) of the disparity in access to basic sanitation services. This result indicates that if all households in the comparison group (rural households) were as wealthy as those in the reference group (urban households), the disparity in access to basic sanitation services would have decreased by 58.17%.

The household heads who are unable to read and write, and lack basic literacy skills, contribute significantly to the rural–urban disparities in accessing basic sanitation services, widening the gap by 16.85% (*β* = 0.04657, 95% CI: 0.02953–0.06360), suggesting that eliminating illiteracy in rural areas would reduce the disparity by 16.85%, indicating a significant positive association.

Differences in primary education attainment between rural and urban households were an endowment factor, which accounted for 0.23% of the disparity in access to basic sanitation services (*β* = 0.00064, 95% CI: 0.00041–0.0.00088). If the same proportion of rural residents attained secondary education as urban households, the gap in access to basic sanitation services would decrease by 3.25% (*β* = −0.00899, 95%CI: −0.01461-0.00337). Conversely, if levels of secondary education attainment were higher in urban areas compared to rural areas, the gap in access would increase by 3.25%.

Additionally, if the same proportion of rural residents had ≤5 family members without under-five children as observed in urban households, the difference between rural and urban areas in access to basic sanitation services would decrease by 5.08% (*β* = −0.01405, 95%CI: −0.02107- −0.00704) and 2.07% (β = −0.00574, 95%CI: −0.01052- −0.00095), respectively.

The increasing disparity in access to basic sanitation services between rural and urban areas was also influenced by differences in pastoralist and agrarian household regions, accounting for 10.10% (*β* = 0.02793, 95% CI: 0.00700–0.04886) and 7.03% (*β* = 0.01942, 95% CI: 0.01171–0.02714), respectively. If households from pastoralist and agrarian regions were to live in urban areas, the disparity in access to basic sanitation services would increase by 10.10 and 7.03%, respectively.

Finally, if rural households had similar access to nearby water sources (within 30 min) as urban households, the gap in access to sanitation services would reduce by 7.91% (*β* = −0.02185, 95% CI: −0.03381- −0.00989).

Approximately 23% of the disparity in access to basic sanitation services between rural and urban areas was attributed to differences in effects or coefficients.

## Discussion

This study aimed to assess rural–urban disparities and the impact of their endowment factors on access to basic sanitation services, using data from the EDHS 2019. The findings reveal that access to basic sanitation services is significantly lower in rural areas compared to urban areas. Through a detailed decomposition analysis, valuable insights into the factors underlying this disparity have been revealed.

The primary driver of this discrepancy is attributed to endowment factors. Specifically, when holding the coefficient effect constant, more than three-quarters of the difference in access to basic sanitation services can be attributed to variations in endowment factors. This highlights the critical role of compositional factors in bridging the rural–urban gap. Urban residents tend to possess a greater abundance of the resources and conditions that facilitate access to basic sanitation services, resulting in their superior access compared to rural residents.

Higher levels of education emerged as a key factor in reducing the disparity in access to basic sanitation services. This finding aligns with previous studies conducted in various regions, which consistently show that urban household heads with higher education are more likely to adopt basic sanitation services than their counterparts (17–22). This tendency can be attributed to greater awareness of health implications, better knowledge of available services, and the perception of sanitation as a marker of status and quality of life.

Urban residents typically benefit from greater economic opportunities, higher living standards, and improved infrastructure, all of which facilitate access to a wider range of basic sanitation services compared to rural residents (23).

This explanation is supported by our study’s descriptive statistics, which show that nearly two-thirds of urban residents, compared with nearly one-third of rural residents, are classified as wealthy, playing a significant role in widening the gap in access to basic sanitation services (8). The literature also supports that higher wealth levels are associated with better access to basic sanitation services (17–22, 24–29).

Additionally, the disparity in access to basic sanitation services between urban and rural areas is significantly influenced by the age of household heads, particularly those between 15 and 35 years (18, 20, 21). This may be because households in this age group in urban areas are more likely to have better access to basic sanitation services, which is driven by higher incomes, better education, and urban infrastructure development.

Disparity in access to basic sanitation services between rural and urban areas is significantly influenced by the presence of under-five children and the total number of family members in households (22). Our analysis suggests that if urban areas had a similar number of under-five children and comparable family sizes to those in rural areas, the gap in sanitation access would decrease by 2.07 and 5.08%, respectively. Notably, rural households tend to have a higher percentage of families with more than five members compared to urban households at 79.82 and 20.18%, respectively. Larger households often experience competition for financial resources, time, and sanitation appliances, making it challenging for household heads to meet the sanitation service requirements.

Moreover, managing the needs of multiple household members requires substantial energy, leading to fatigue and stress, which can hinder a household’s ability to ensure access to basic sanitation services. Consequently, households with fewer than five members are more likely to have access to basic sanitation services. This finding is in line with the studies conducted in Jimma, Tanzania, and Nigeria (17, 22, 25).

The disparity in access to basic sanitation services between rural and urban areas is also significantly influenced by differences in household regions (10, 17, 24, 29–31) and the proximity of water sources (19–21, 24). The possible reasons could include differences in infrastructure development, governmental prioritization of urban areas, disparities in resource allocation, environmental conditions, and varying population densities, all of which affect access to sanitation services.

Behavioral or effect factors accounted for 22.1% of the disparities in access to basic sanitation services between urban and rural residents. The coefficients for unexplained factors indicated that changes in covariates aimed at increasing access to basic sanitation services may lead to only marginal improvements in rural areas, which are insignificant compared to urban areas. This suggests that while behavioral changes are important, none of the coefficients for unexplained factors were significant in explaining the gap in access to basic sanitation services between rural and urban areas.

### Strengths and limitations of the study

The current decomposition analysis study provides valuable insights into the complex factors contributing to rural–urban disparities in access to basic sanitation services. These findings can assist environmental and public health practitioners in designing targeted interventions aimed at reducing the gap and improving overall health outcomes. Additionally, the results are representative of the source population, as the data are drawn from a nationwide sample.

However, this study has limitations. First, the findings are based on cross-sectional data, which restricts the ability to establish causal relationships. Second, the study relied on self-reported data that may be subject to recall bias or social desirability bias. However, efforts were made to minimize these biases through rigorous data collection procedures.

Finally, it is important to acknowledge that the study did not explore all possible factors influencing access to basic sanitation services. Specifically, the analysis did not explicitly examine other relevant variables, such as cultural norms, infrastructure availability, and policy interventions. Future research could adopt a more comprehensive approach to examine these additional factors and their impact on sanitation access.

## Conclusion

There is a significant disparity in access to basic sanitation services between rural and urban residents, with the majority of this gap explained by the endowment effect. Key factors contributing to this discrepancy include the age of household heads (15–35 years), household heads who are unable to read and write, attainment of primary and secondary education, the presence of under-five children, households with fewer than five family members, residence in pastoralist and agrarian regions, reliance on water sources located more than 30 min away, and poverty. These factors were found to be significant in explaining the differences in access to basic sanitation services between urban and rural regions.

Therefore, it is recommended to implement comprehensive sanitation and hygiene education programs, ensure sustainable sanitation infrastructure for rural communities, and promote community-based water source management. Additionally, developing affordable sanitation products, integrating sanitation education into maternal and child health services, and establishing effective community-led total sanitation services are crucial. Enhancing the capacity of local governments, fostering public-private partnerships, and implementing robust monitoring systems to track the progress and impact of sanitation interventions are also essential steps in improving access to basic sanitation services.

## Data Availability

The original contributions presented in the study are included in the article/supplementary material. Further inquiries can be directed to the corresponding author.
